# Molecular and Electrophysiological Mechanisms Underlying Cardiac Arrhythmogenesis in Diabetes Mellitus

**DOI:** 10.1155/2016/2848759

**Published:** 2016-08-23

**Authors:** Gary Tse, Eric Tsz Him Lai, Vivian Tse, Jie Ming Yeo

**Affiliations:** ^1^Department of Medicine and Therapeutics, The Chinese University of Hong Kong, Shatin, Hong Kong; ^2^School of Biomedical Sciences, Li Ka Shing Faculty of Medicine, University of Hong Kong, Hong Kong; ^3^Department of Physiology, McGill University, Montreal, QC, Canada H3G 1Y6; ^4^School of Medicine, Imperial College London, London SW7 2AZ, UK

## Abstract

Diabetes is a common endocrine disorder with an ever increasing prevalence globally, placing significant burdens on our healthcare systems. It is associated with significant cardiovascular morbidities. One of the mechanisms by which it causes death is increasing the risk of cardiac arrhythmias. The aim of this article is to review the cardiac (ion channel abnormalities, electrophysiological and structural remodelling) and extracardiac factors (neural pathway remodelling) responsible for cardiac arrhythmogenesis in diabetes. It is concluded by an outline of molecular targets for future antiarrhythmic therapy for the diabetic population.

## 1. Introduction

**Figure 1:**
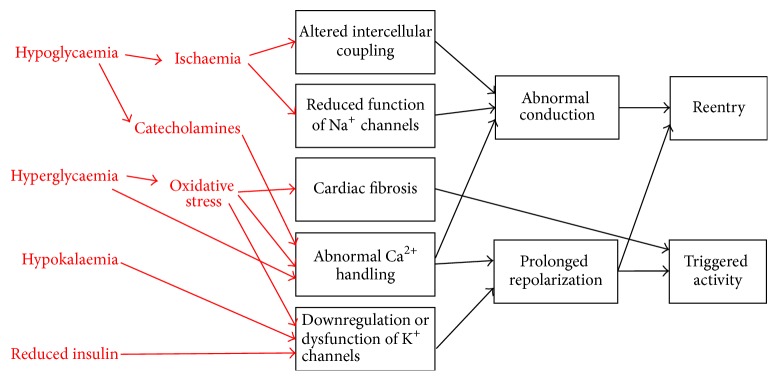
Both conduction and repolarization abnormalities promote arrhythmogenesis in diabetes.

**Figure 2:**
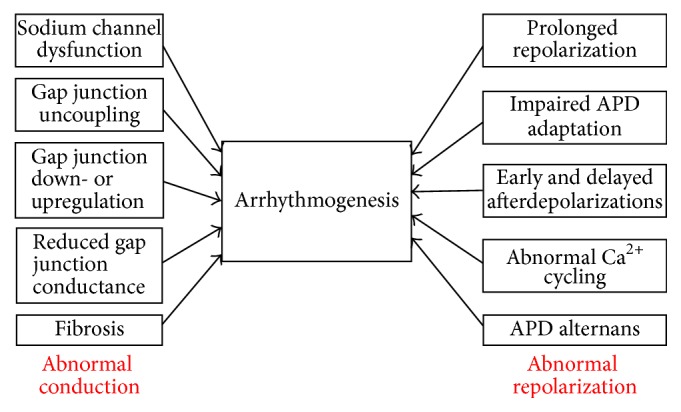
Cardiac and extracardiac factors responsible for promoting arrhythmogenesis in diabetes.

**Figure 3:**
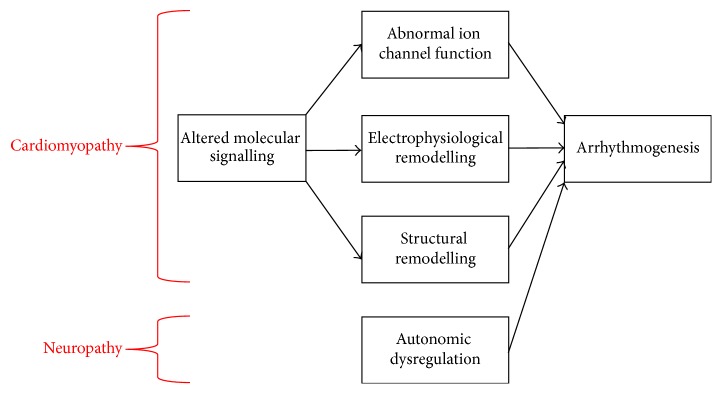


Cardiometabolic disorders place significant burdens on the healthcare system worldwide [1]. Their prevalence has been rising over the past decades due to an aging population and an increasing level of obesity [2, 3]. Diabetes mellitus is an endocrine disorder characterized by reduced insulin production (type 1) or increased insulin resistance (type 2), leading to hyperglycaemia. There is increasing evidence that diabetes increases the risk of cardiac arrhythmias. This involves abnormalities in action potential conduction or repolarization (Figures 1 and 2), due to a complex interplay of ion channel abnormalities and electrophysiological remodelling superimposed upon a cardiomyopathic process together with autonomic dysregulation (Figure 3). Some of these findings are derived from experiments performed in animal models, which have been proven extremely useful for dissecting the molecular mechanisms responsible for arrhythmic phenotypes [4]. In this review, the pathophysiology underlying cardiac arrhythmias in diabetes mellitus is explored in detail, followed by an outline of potential therapeutic targets for reducing arrhythmic risk and sudden death in diabetic patients.

## 2. Arrhythmogenic Mechanisms in Diabetes Mellitus

The common arrhythmogenic mechanism is reentry, which occurs when an action potential fails to extinguish itself and reactivates a region that has recovered from refractoriness. This can arise from abnormalities in conduction or repolarization or both [5]. Circus reentry requires three prerequisites: (i) conduction velocity (CV) which must be sufficiently slowed so that the tissue ahead of the action potential (AP) wavefront remains excitable, (ii) unidirectional conduction block which must be present to prevent waves from self-extinguishing when they collide, and (iii) an obstacle around which an AP can circulate [6]. This need not be a structural defect but can be a functional core of refractory tissue, which may arise dynamically from ectopic activity [7]. Repolarization abnormalities can result in early or delayed afterdepolarizations (EADs and DADs), which can initiate triggered activity when their magnitudes are sufficiently large to reach the threshold potential for sodium channel reactivation. They can also increase the dispersion of repolarization, promoting unidirectional conduction block and reentry. In diabetes mellitus, arrhythmogenesis can be due to the following mechanisms. Abnormalities in conduction are mediated by myocardial ischaemia [8] or in repolarization [9, 10] by ion channel dysfunction, increased adrenergic drive, and calcium overload [11]. These abnormalities are superimposed upon a cardiomyopathy, in which the structural changes also predispose to arrhythmias. Extracardiac abnormalities, for example, neural pathway remodelling, can further promote arrhythmogenesis [12]. Ventricular arrhythmias are thought to underlie sudden cardiac death (SCD) in type 2 diabetic patients and also the “dead-in-bed syndrome” observed in otherwise young healthy adults with type 1 diabetes [13].

## 3. Abnormal Conduction

CV depends upon sodium channel activation followed by electrotonic spread of the ionic currents via gap junctions, which are electrical coupling pathways located between adjacent cardiomyocytes [14]. Each gap junction is made of two connexons, and each connexon is a hexamer of connexins (Cx). Altered gap junction expression or function can produce conduction abnormalities and in turn predispose to reentrant excitation. Protein kinase C- (PKC-) mediated phosphorylation, a calcium-dependent process, at serine 368 of Cx43, has been linked to reduced gap junction conductance [15, 16]. Dephosphorylation of gap junctions results in their uncoupling [17] and lateralization [18, 19]. There is consistent evidence demonstrating altered gap junction function or expression in different experimental models of diabetes. Thus, in transgenic mice with cardiac-specific overexpression of peroxisome proliferator-activated receptor *γ* 1 (PPAR*γ*1) modelling human diabetes, reduced Cx43 expression without alterations in CV was observed [20]. This may increase anisotropy and higher likelihood of reentry. In streptozotocin- (STZ-) induced diabetic rats, expression levels of Cx40, 43 and 45 in the SA node, are significantly increased, which were associated with SA conduction delay [21]. This can be explained by increased expression levels of Cx45, which has the lowest unitary conductance and whose expression reduces CV. In both atria and ventricles of the same model, Cx43 phosphorylation was decreased because of reduced PKC*ε* expression [22]; Cx43 was upregulated in the atria, whereas its expression level was unchanged in the ventricles [23]. Furthermore, the lack of insulin signalling can lead to reduced CV of propagating APs.

Myocardial fibrosis is increasingly recognized to be a pathogenic factor in diabetic cardiomyopathy [24]. Fibrosis resulting from fibroblast activation is mediated by growth factors, such as transforming growth factor-*β* [25]. This produces conduction abnormalities via two mechanisms: (i) reduced coupling between cardiomyocytes, leading to increased axial resistance; (ii) increased coupling between fibroblast and cardiomyocyte, increasing membrane capacitance [26]. Both mechanisms lead to a decrease in CV. Cardiac magnetic resonance (CMR) with late gadolinium enhancement is used for the diagnosis and monitoring of cardiomyopathy [27–29] and is potentially useful for examining fibrosis in diabetic cardiomyopathy.

Hypoglycemic episodes are associated with myocardial ischaemia [8], which may predispose to ventricular arrhythmias by producing conduction defects via the following mechanisms [14]. Ischaemia results in ATP depletion, metabolic switching to anaerobic glycolysis, extracellular H^+^ accumulation, and intracellular Ca^2+^ overload. Cytosolic Ca^2+^ binds to the conserved C2 domain of PKC, thereby activating it [30]. There are several downstream targets of PKC. Firstly, PKC phosphorylates the serine residue at 1505 position of the sodium channel inactivation gate between domains III and IV, which decreases *I*_Na_ [31]. Secondly, it also phosphorylates connexins (Cx) 43 at serine 368, reducing gap junction conductance [15, 16]. Ca^2+^ overload is also associated with dephosphorylation of gap junctions [32], resulting in their uncoupling [17] and lateralization [18, 19]. Thus, myocardial ischaemia secondary to hypoglycaemia reduces CV and increases dispersion of conduction, predisposing to reentrant excitation.

## 4. Abnormal Repolarization

Action potential repolarization has two phases: (i) early rapid repolarization resulting from the activation of the fast and slow transient outward potassium currents, *I*_to,f_ and *I*_to,s_, and (ii) prolonged plateau resulting from a balance between the inward currents mediated by the voltage-gated L-type calcium channel (LTCC, *I*_Ca,L_) and sodium-calcium exchanger (*I*_NCX_) and the outward currents mediated by the voltage-gated delayed rectifier potassium channels (*I*_K_: rapid and slow currents, *I*_Kr_ and *I*_Ks_) [33]. There is also contribution from the inward rectifying current (*I*_K1_). Of these, the human ether-à-go-go-related gene (HERG) K^+^ channel is the major component of delayed rectifier K^+^ current [34].

In diabetes mellitus, prolongations in action potential durations (APDs) are due to several mechanisms. The lack of insulin signalling resulted in electrophysiological remodelling: *I*_to_ is reduced as a result of reduced expression of Kv4.2 and KChiP2 genes [35]. This current is posttranslationally regulated by a number of different kinases. For example, the p90 ribosomal S6 kinase (p90RSK) is a serine/threonine kinase with N- and C-terminal kinase domains. Reactive oxygen species (ROS), which are raised in diabetes [36], increases the activity of p90RSK and reduced the activity of *I*_to,f_, *I*_K,slow_, and *I*_SS_ channels [37]. Moreover, transgenic mice with cardiac-specific overexpression of peroxisome proliferator-activated receptor *γ* 1 (PPAR*γ*1) showed abnormal lipid accumulation in cardiomyocytes and reduced expression as well as function of *I*_to,f_ and *I*_K,slow_[20]. The Rad (Ras associated with diabetes) protein is implicated in diabetes: in its dominant negative mutant, LTCC was upregulated [38]. Together, increased inward currents and decreased outward currents lead to prolonged ventricular repolarization. Conversely, genetic mutations of key ion channel genes causing prolonged ventricular repolarization can also lead to diabetes. For example, mutations in KCNE2 are responsible for long QT syndrome type 5. Whole-transcript transcriptomics demonstrated that KCNE2^−/−^ mice additionally showed diabetes mellitus, hypercholesterolemia, and elevated angiotensin II levels [39]. Hypoglycaemia causes intracellular depletion of ATP in cardiomyocytes and hyperglycaemia increases the production of reactive oxygen species (ROS), both leading to HERG channel dysfunction [40]. K_ATP_ channels are thought to provide a link between cellular energy status and membrane electrophysiology. They are normally inhibited by ATP and activated by ADP. During ischaemia, there are ATP depletion and ADP accumulation, activating *I*_K,ATP_ and promoting APD shortening [41]. In diabetes, initial APD shortening is also observed but this becomes fully reversed in a time-dependent manner. This failure of APD adaptation, when accompanied by increased adrenergic drive, can engage in steep APD restitution, in turn leading to the production of arrhythmogenic APD alternans [7].

Hypoglycaemia is also associated with another cause of delayed repolarization, hypokalaemia [42, 43], which arises from insulin therapy or increased adrenergic drive [44, 45]. Hypokalaemia inhibits *I*_K1_, thereby prolonging APDs and causing L-type Ca^2+^ channel reactivation [46]. This then leads to early afterdepolarizations (EADs) and consequent triggered activity [47]. Hypokalaemia also preferentially prolongs epicardial APDs and leaving endocardial APDs unchanged, increasing the transmural repolarization gradient [47]. In combination with reduced effective refractory periods (ERPs), excitation wavelength (conduction velocity (CV) × ERP) is reduced. Furthermore, increased steepness of APD restitution results in the development of APD alternans [48] and in turn in wavebreak, conduction block, and initiation and maintenance of reentrant activity [7, 49].

Hypoglycaemia also increases adrenergic drive with the following proarrhythmic consequences [50]. Firstly, the release of catecholamines leads to abnormal Ca^2+^ cycling and intracellular Ca^2+^ accumulation. This in turn stimulates spontaneous Ca^2+^ release from the sarcoplasmic reticulum, thereby activating three calcium-sensitive currents: the nonselective cationic current, *I*_NS_, the sodium-calcium exchange current, *I*_NCX_, and the calcium-activated chloride current, *I*_Cl,Ca_. Thus, such inward currents observed during phase 4 of the action potential lead to delayed afterdepolarizations (DADs), eliciting triggered activity.

Abnormal Ca^2+^ dynamics have been implicated in diabetes. For example, cardiomyocytes of leptin-deficient ob/ob mice showed reduced amplitudes of Ca^2+^ transients, and insulin elicited extra transients via inositol 1,4,5-trisphosphate (IP_3_) signalling and impaired mitochondrial Ca^2+^ handling [51]. Furthermore, decreases in DAG-mediated nonselective cation currents were associated with reduced TRPC3 expression at the plasma membrane, which increases Ca^2+^ influx [52]. Dysregulation of the type 2 ryanodine receptor (RyR2) has been detected in a STZ-induced diabetes rat model, in which increased frequency of Ca^2+^ sparks with reduced amplitudes was associated with increased sensitivity to Ca^2+^ activation and dyssynchronous Ca^2+^ release [53, 54]. Abnormal RyR2 gating mechanism may arise from increased phosphorylation by protein kinase A (PKA, serine 2808) and Ca^2+^/calmodulin-dependent protein kinase II (CaMKII, serine 2808 and serine 2814) [55–57], as well as oxidation by ROS and reactive carbonyl species (RCS), which are increased in diabetes [58–60]. Uncontrolled hyperglycaemia can lead to activation of CaMKII and subsequent Ca^2+^ release from the SR [61]. Dyssynchronous Ca^2+^ release can be explained by remodelling of the transverse tubular system, whereby RyR2 become orphaned when they are decoupled from LTCCs [62]. Interestingly, catecholaminergic polymorphic ventricular tachycardia (CPVT) is caused by RyR2 mutation, and patients suffering from this condition are also prone to impaired glucose homeostasis and insulin secretion [63]. It would be interesting to determine whether diabetic patients with acquired dysfunction in RyR2 develop bidirectional VT classically associated with CPVT.

Moreover, diabetes mellitus is an independent risk factor for atrial fibrillation, yet the underlying physiological mechanisms are incompletely understood. It may involve ion channel remodelling in the atria. For example, the small conductance Ca^2+^-activated K^+^ (SK) channels contribute to atrial repolarization. SK2 and SK3 isoforms are downregulated, leading to APD prolongation [64]. Normally, SK channels do not play a role in ventricular repolarization. In heart failure, SK currents and ion channel expression can be upregulated and become more sensitive to Ca^2+^ modulation, potentially leading to ventricular arrhythmias [65]. Altered expression of SK channels in the ventricles may play a role in diabetes but this remains to be tested experimentally.

## 5. Diabetic Cardiomyopathy: Cardiac Electrophysiological and Structural Remodelling with Superimposed Autonomic Dysregulation

Diabetic cardiomyopathy is characterized by diastolic dysfunction with preserved systolic function, findings that are similarly observed in genetically modified, leptin receptor deficient, diabetic db/db mice on echocardiography [66, 67]. Cardiac magnetic resonance imaging is excellent for characterizing structural abnormalities, such as areas of fibrosis by late gadolinium enhancement [27–29]. Afferent and efferent neural pathways normally regulate inotropic, lusitropic, chronotropic, and dromotropic responses of the heart. In diabetes, these can become dysregulated with impaired baroreceptor control of heart rate [68]. Reduced heart rate variability (HRV) has long been associated with increased mortality [69]. In diabetes, a reduction in HRV was associated with increased incidence of inducible VT by programmed electrical stimulation [70]. Electrophysiological modelling is likely to be an early event, appearing before structural abnormalities. Thus, STZ-induced diabetic rats showed decreases in both maximal transport capacity of SERCA2a and RyR2 conductance, associated with impairment of both inotropic and lusitropic responses in response to adrenergic stimulation [71]. This finding differs from human findings with impaired positive inotropic response with preservation of positive lusitropic effects of beta-adrenoceptor stimulation [72].

Brady-arrhythmias in the form of sinoatrial (SA) and atrioventricular (AV) nodal blocks are seen in diabetes [73, 74]. Sinoatrial node (SAN) dysfunction was demonstrated in db/db mice, which demonstrated prolonged SAN recovery time [66]. These mice showed no significant differences in conduction intervals and wave amplitudes compared to control mice. By contrast, sinus tachycardia at rest has been associated with excessive mortality in diabetic patients [75]. This may be related to autonomic dysregulation, with increased adrenergic drive with or without impairment of parasympathetic response. Thus, in Akita diabetic mice, the SA node is less responsive to acetylcholine because of a reduction in acetylcholine-activated K^+^ current (*I*_K,ACh_), which is due to altered phosphoinositide 3-kinase (PI3K) signalling [76].

Some aspects of altered cardiac electrophysiology in diabetes do not arise from abnormalities in the heart itself, but instead from neural pathways innervating it. Thus, in STZ-induced diabetic mice, both baroreflex tachycardia and bradycardia were blunted. This was associated with remodelling of the baroreceptor circuitry, in which the sizes of cardiac ganglia and ganglionic principal neurons were decreased. In a different model, the OVE26 diabetic mice showed neural degeneration in the nucleus ambiguus, which is one of the two brainstem nuclei innervating the cardiac ganglia [77]. Furthermore, altered balance between chemoattractants (e.g., nerve growth factor) and chemorepellants (Sema3a) leads to disruptions in innervation pattern, precipitating arrhythmias, and sudden death [78].

## 6. Clinical Relevance and Future Therapies

Traditional agents used for treatment of diabetes or associated comorbidities such as hypertension have been shown to exert cardiac protective effects in diabetes by previously unknown mechanisms. Thus, for example, in the STZ-induced diabetic rat model, *I*_to_ and *I*_SS_ are downregulated and the cardiac renin-angiotensin system is activated. Experimental evidence has demonstrated augmentation of both currents by the antihypertensive angiotensin II receptor blockers [79]. The ACE inhibitor enalapril [80] and angiotensin II receptor blocker losartan [81] were also shown to exert antifibrotic effects in hypertension and may have similar cardioprotective effects in diabetes by similar mechanisms. The antifibrotic hormone relaxin could be delivered using adenoviruses [82] and may reverse fibrosis in diabetic cardiomyopathy. Ion channels represent an attractive target for managing arrhythmic complications of diabetes mellitus (Table 1). Novel agents such as late sodium current blockers [83] and gap junction openers [49] can be used to reduce abnormal repolarization and conduction, respectively. Alternatively, gap junction inhibitors can prolong effective refractory periods and exert antiarrhythmic effects [47]. Paradoxically, mild gap junction uncoupling could improve the safety margin of conduction and increase CV, removing unidirectional conduction blocks and converting these into bilateral conduction. Their use in diabetes warrants future exploration. Ryanodine receptor stabilizers have the potential to normalize Ca^2+^ handling in diabetes, which remains to be tested [84]. However, caution must be exercised to screen for deleterious, ventricular proarrhythmic effects. K_ATP_ channels play a role in not only insulin secretion but also cardiac repolarization. Whilst the K_ATP_ channel activators have been used to increase insulin release, they have the potential to cause life-threatening ventricular arrhythmias, especially in a subset of patients with ischaemic complications. In diabetes, mitochondrial K_ATP_ channel activation in cardiomyocytes by dioxide led to impaired APD adaptation, which promoted the occurrence of VT [85]. Future efforts therefore require an integrated approach by computation modelling, where effects of drugs on complex spatiotemporal properties of cardiac dynamics are tested to reduce the likelihood of life-threatening side effects. Animal models will be useful for studying arrhythmogenic mechanisms and provide a platform for assessing the efficacy of pharmacological therapy with translational applications [86–88].

## Figures and Tables

**Table 1:** 

Molecular target	Mechanism of action	References
Gap junction inhibitors	Increase refractory period Improve conduction	[47]

Gap junction openers	Increase conduction velocity and decrease heterogeneity in repolarization or refractoriness	[49]

Late sodium channel blockers	Inhibit afterdepolarizations	[83]

Ryanodine receptor stabilizers	Decrease heterogeneity in Ca^2+^ transients and inhibit afterdepolarizations	[84]

Antifibrotic agents	Reduce cardiac fibrosis	[82]

## References

[1] Choy L., Yeo J. M., Tse V., Chan S. P., Tse G. (2016). Cardiac disease and arrhythmogenesis: mechanistic insights from mouse models. *IJC Heart & Vasculature*.

[2] Chan R. S. M., Woo J. (2010). Prevention of overweight and obesity: how effective is the current public health approach. *International Journal of Environmental Research and Public Health*.

[3] Yi Wong E. L., Woo J., Hui E., Chan C., Chan W. L. S., Cheung A. W. L. (2011). Primary care for diabetes mellitus: perspective from older patients. *Patient Preference and Adherence*.

[4] Tse G., Wong S. T., Tse V., Yeo J. M. (2016). Monophasic action potential recordings: which is the recording electrode?. *Journal of Basic and Clinical Physiology and Pharmacology*.

[5] Tse G., Lai E. T. H., Yeo J. M. (2016). Mechanisms of electrical activation and conduction in the gastrointestinal system: lessons from cardiac electrophysiology. *Frontiers in Physiology*.

[6] Tse G., Lai E. T., Lee A. P., Yan B. P., Wong S. H. (2016). Electrophysiological mechanisms of gastrointestinal arrhythmogenesis: lessons from the heart. *Frontiers in Physiology*.

[7] Tse G., Wong S. T., Tse V., Lee Y. T., Lin H. Y., Yeo J. M. (2016). Cardiac dynamics: alternans and arrhythmogenesis. *Journal of Arrhythmia*.

[8] Desouza C., Salazar H., Cheong B., Murgo J., Fonseca V. (2003). Association of hypoglycemia and cardiac ischemia: a study based on continuous monitoring. *Diabetes Care*.

[9] Marques J. L. B., George E., Peacey S. R. (1997). Altered ventricular repolarization during hypoglycaemia in patients with diabetes. *Diabetic Medicine*.

[10] Bolognesi R., Tsialtas D., Bolognesi M. G., Giumelli C. (2011). Marked sinus bradycardia and QT prolongation in a diabetic patient with severe hypoglycemia. *Journal of Diabetes and its Complications*.

[11] Tse G., Lai E. T. H., Yeo J. M., Yan B. P. (2016). Electrophysiological mechanisms of Bayés syndrome: insights from clinical and mouse studies. *Frontiers in Physiology*.

[12] Coumel P. (1993). Cardiac arrhythmias and the autonomic nervous system. *Journal of Cardiovascular Electrophysiology*.

[13] Tattersall R. B., Gill G. V. (1991). Unexplained deaths of Type 1 diabetic patients. *Diabetic Medicine*.

[14] Tse G., Yeo J. M. (2015). Conduction abnormalities and ventricular arrhythmogenesis: the roles of sodium channels and gap junctions. *IJC Heart & Vasculature*.

[15] Moreno A. P., Sáez J. C., Fishman G. I., Spray D. C. (1994). Human connexin43 gap junction channels: regulation of unitary conductances by phosphorylation. *Circulation Research*.

[16] Kwak B. R., Hermans M. M. P., De Jonge H. R., Lohmann S. M., Jongsma H. J., Chanson M. (1995). Differential regulation of distinct types of gap junction channels by similar phosphorylating conditions. *Molecular Biology of the Cell*.

[17] Beardslee M. A., Lerner D. L., Tadros P. N. (2000). Dephosphorylation and intracellular redistribution of ventricular connexin43 during electrical uncoupling induced by ischemia. *Circulation Research*.

[18] Smith J. H., Green C. R., Peters N. S., Rothery S., Severs N. J. (1991). Altered patterns of gap junction distribution in ischemic heart disease: an immunohistochemical study of human myocardium using laser scanning confocal microscopy. *American Journal of Pathology*.

[19] Lampe P. D., TenBroek E. M., Burt J. M., Kurata W. E., Johnson R. G., Lau A. F. (2000). Phosphorylation of connexin43 on serine368 by protein kinase C regulates gap junctional communication. *The Journal of Cell Biology*.

[20] Morrow J. P., Katchman A., Son N.-H. (2011). Mice with cardiac overexpression of peroxisome proliferator-activated receptor *γ* have impaired repolarization and spontaneous fatal ventricular arrhythmias. *Circulation*.

[21] Howarth F. C., Nowotny N., Zilahi E., El Haj M. A., Lei M. (2007). Altered expression of gap junction connexin proteins may partly underlie heart rhythm disturbances in the streptozotocin-induced diabetic rat heart. *Molecular and Cellular Biochemistry*.

[22] Lin H., Mitasikova M., Dlugosova K. (2008). Thyroid hormones suppress *ε*-PKC signalling, down-regulate connexin-43 and increase lethal arrhythmia susceptibility in non-diabetic and diabetic rat hearts. *Journal of Physiology and Pharmacology*.

[23] Mitašíková M., Lin H., Soukup T., Imanaga I., Tribulová N. (2009). Diabetes and thyroid hormones affect connexin-43 and PKC-*ε* expression in rat heart atria. *Physiological Research*.

[24] Asbun J., Villarreal F. J. (2006). The pathogenesis of myocardial fibrosis in the setting of diabetic cardiomyopathy. *Journal of the American College of Cardiology*.

[25] Leask A. (2010). Potential therapeutic targets for cardiac fibrosis: TGF*β*, angiotensin, endothelin, CCN2, and PDGF, partners in fibroblast activation. *Circulation Research*.

[26] Miragoli M., Gaudesius G., Rohr S. (2006). Electrotonic modulation of cardiac impulse conduction by myofibroblasts. *Circulation Research*.

[27] Tse G., Ali A., Alpendurada F., Prasad S., Raphael C. E., Vassiliou V. (2015). Tuberculous constrictive pericarditis. *Research in Cardiovascular Medicine*.

[28] Tse G., Ali A., Prasad S. K., Vassiliou V., Raphael C. E. (2015). Atypical case of post-partum cardiomyopathy: an overlap syndrome with arrhythmogenic right ventricular cardiomyopathy?. *BJR: Case Reports*.

[29] Vassiliou V., Chin C., Perperoglou A. (2014). 93Ejection fraction by cardiovascular magnetic resonance predicts adverse outcomes post aortic valve replacement. *Heart*.

[30] Luo J.-H., Weinstein I. B. (1993). Calcium-dependent activation of protein kinase C: the role of the C2 domain in divalent cation selectivity. *The Journal of Biological Chemistry*.

[31] Qu Y., Rogers J. C., Tanada T. N., Catterall W. A., Scheuer T. (1996). Phosphorylation of S1505 in the cardiac Na+ channel inactivation gate is required for modulation by protein kinase C. *Journal of General Physiology*.

[32] Huang X.-D., Sandusky G. E., Zipes D. P. (1999). Heterogeneous loss of connexin43 protein in ischemic dog hearts. *Journal of Cardiovascular Electrophysiology*.

[33] Carmeliet E. (1999). Cardiac ionic currents and acute ischemia: from channels to arrhythmias. *Physiological Reviews*.

[34] Sanguinetti M. C., Jiang C., Curran M. E., Keating M. T. (1995). A mechanistic link between an inherited and an acquird cardiac arrthytmia: HERG encodes the I_Kr_ potassium channel. *Cell*.

[35] Lopez-Izquierdo A., Pereira R. O., Wende A. R., Punske B. B., Dale Abel E., Tristani-Firouzi M. (2014). The absence of insulin signaling in the heart induces changes in potassium channel expression and ventricular repolarization. *American Journal of Physiology—Heart and Circulatory Physiology*.

[36] Tse G., Yan B. P., Chan Y. W. F., Tian X. Y., Huang Y. (2016). Reactive oxygen species, endoplasmic reticulum stress and mitochondrial dysfunction: the link with cardiac arrhythmogenesis. *Frontiers in Physiology*.

[37] Lu Z., Abe J.-I., Taunton J. (2008). Reactive oxygen species-induced activation of p90 ribosomal s6 kinase prolongs cardiac repolarization through inhibiting outward K^+^ channel activity. *Circulation Research*.

[38] Yada H., Murata M., Shimoda K. (2007). Dominant negative suppression of Rad leads to QT prolongation and causes ventricular arrhythmias via modulation of L-type Ca^2+^ channels in the heart. *Circulation Research*.

[39] Hu Z., Kant R., Anand M. (2014). Kcne2 deletion creates a multisystem syndrome predisposing to sudden cardiac death. *Circulation: Cardiovascular Genetics*.

[40] Zhang Y., Han H., Wang J., Wang H., Yang B., Wang Z. (2003). Impairment of human ether-à-go-go-related gene (HERG) K^+^ channel function by hypoglycemia and hyperglycemia: similar phenotypes but different mechanisms. *The Journal of Biological Chemistry*.

[41] Xie C., Hu J., Motloch L. J., Karam B. S., Akar F. G. (2015). The classically cardioprotective agent diazoxide elicits arrhythmias in type 2 diabetes mellitus. *Journal of the American College of Cardiology*.

[42] Heller S. R., Robinson R. T. C. E. (2000). Hypoglycaemia and associated hypokalaemia in diabetes: mechanisms, clinical implications and prevention. *Diabetes, Obesity and Metabolism*.

[43] Christensen T. F., Bækgaard M., Dideriksen J. L. (2009). A physiological model of the effect of hypoglycemia on plasma potassium. *Journal of Diabetes Science and Technology*.

[44] Fisher B. M., Thomson I., Hepburn D. A., Frier B. M. (1991). Effects of adrenergic blockade on serum potassium changes in response to acute insulin-induced hypoglycemia in nondiabetic humans. *Diabetes Care*.

[45] Petersen K. G., Schluter K. J., Kerp L. (1982). Regulation of serum potassium during insulin-induced hypoglycemia. *Diabetes*.

[46] January C. T., Riddle J. M. (1989). Early afterdepolarizations: mechanism of induction and block. A role for L-type Ca2+ current. *Circulation Research*.

[47] Tse G., Tse V., Yeo J. M., Sun B., Talkachova A. (2016). Atrial anti-arrhythmic effects of heptanol in Langendorff-perfused mouse hearts. *PLoS ONE*.

[48] Tse G., Wong S. T., Tse V., Yeo J. (2016). Restitution analysis of alternans using dynamic pacing and its comparison with S1S2 restitution in heptanol-treated, hypokalaemic Langendorff-perfused mouse hearts. *Biomedical Reports*.

[49] Hsieh Y.-C., Lin J.-C., Hung C.-Y. (2016). Gap junction modifier rotigaptide decreases the susceptibility to ventricular arrhythmia by enhancing conduction velocity and suppressing discordant alternans during therapeutic hypothermia in isolated rabbit hearts. *Heart Rhythm*.

[50] Goldfien A., Moore R., Zileli S., Havens L. L., Boling L., Thorn G. W. (1961). Plasma epinephrine and norepinephrine levels during insulin-induced hypoglycemia in man. *The Journal of clinical endocrinology & metabolism*.

[51] Fauconnier J., Lanner J. T., Zhang S. J. (2005). Insulin and inositol 1,4,5-trisphosphate trigger abnormal cytosolic Ca^2+^ transients and reveal mitochondrial Ca^2+^ handling defects in cardiomyocytes of *ob/ob* mice. *Diabetes*.

[52] Fauconnier J., Lanner J. T., Sultan A. (2007). Insulin potentiates TRPC3-mediated cation currents in normal but not in insulin-resistant mouse cardiomyocytes. *Cardiovascular Research*.

[53] Shao C.-H., Rozanski G. J., Patel K. P., Bidasee K. R. (2007). Dyssynchronous (non-uniform) Ca^2+^ release in myocytes from streptozotocin-induced diabetic rats. *Journal of Molecular and Cellular Cardiology*.

[54] Shao C.-H., Wehrens X. H. T., Wyatt T. A. (2009). Exercise training during diabetes attenuates cardiac ryanodine receptor dysregulation. *Journal of Applied Physiology*.

[55] Hain J., Onoue H., Mayrleitner M., Fleischer S., Schindler H. (1995). Phosphorylation modulates the function of the calcium release channel of sarcoplasmic reticulum from cardiac muscle. *The Journal of Biological Chemistry*.

[56] Wehrens X. H. T., Lehnart S. E., Reiken S. R., Marks A. R. (2004). Ca^2+^/calmodulin-dependent protein kinase II phosphorylation regulates the cardiac ryanodine receptor. *Circulation Research*.

[57] Witcher D. B., Kovacs R. J., Schulman H., Cefali D. C., Jones L. R. (1991). Unique phosphorylation site on the cardiac ryanodine receptor regulates calcium channel activity. *The Journal of Biological Chemistry*.

[58] Bidasee K. R., Nallani K., Besch H. R., Deniz Dincer U. (2003). Streptozotocin-induced diabetes increases disulfide bond formation on cardiac ryanodine receptor (RyR2). *Journal of Pharmacology and Experimental Therapeutics*.

[59] Eager K. R., Roden L. D., Dulhunty A. F. (1997). Actions of sulfhydryl reagents on single ryanodine receptor Ca^2+^-release channels from sheep myocardium. *The American Journal of Physiology*.

[60] Xu L., Eu J. P., Meissner G., Stamler J. S. (1998). Activation of the cardiac calcium release channel (ryanodoine receptor) by poly-S-nitrosylation. *Science*.

[61] Erickson J. R., Pereira L., Wang L. (2013). Diabetic hyperglycaemia activates CaMKII and arrhythmias by O-linked glycosylation. *Nature*.

[62] Song L.-S., Sobie E. A., McCulle S., Lederer W. J., Balke C. W., Cheng H. (2006). Orphaned ryanodine receptors in the failing heart. *Proceedings of the National Academy of Sciences of the United States of America*.

[63] Santulli G., Pagano G., Sardu C. (2015). Calcium release channel RyR2 regulates insulin release and glucose homeostasis. *The Journal of Clinical Investigation*.

[64] Yi F., Ling T.-Y., Lu T. (2015). Down-regulation of the small conductance calcium-activated potassium channels in diabetic mouse atria. *The Journal of Biological Chemistry*.

[65] Chang P.-C., Chen P.-S. (2015). SK channels and ventricular arrhythmias in heart failure. *Trends in Cardiovascular Medicine*.

[66] Soltysinska E., Speerschneider T., Winther S. V., Thomsen M. B. (2014). Sinoatrial node dysfunction induces cardiac arrhythmias in diabetic mice. *Cardiovascular Diabetology*.

[67] Basso C., Calabrese F., Angelini A., Carturan E., Thiene G. (2013). Classification and histological, immunohistochemical, and molecular diagnosis of inflammatory myocardial disease. *Heart Failure Reviews*.

[68] Fukuda K., Kanazawa H., Aizawa Y., Ardell J. L., Shivkumar K. (2015). Cardiac innervation and sudden cardiac death. *Circulation Research*.

[69] Lombardi F. (2000). Chaos theory, heart rate variability, and arrhythmic mortality. *Circulation*.

[70] Rajab M., Jin H., Welzig C. M. (2013). Increased inducibility of ventricular tachycardia and decreased heart rate variability in a mouse model for type 1 diabetes: effect of pravastatin. *American Journal of Physiology—Heart and Circulatory Physiology*.

[71] Ligeti L., Szenczi O., Prestia C. M. (2006). Altered calcium handling is an early sign of streptozotocin-induced diabetic cardiomyopathy. *International Journal of Molecular Medicine*.

[72] Amour J., Loyer X., Michelet P., Birenbaum A., Riou B., Heymes C. (2008). Preservation of the positive lusitropic effect of *β*-adrenoceptors stimulation in diabetic cardiomyopathy. *Anesthesia & Analgesia*.

[73] Hasslacher C., Wahl P. (1977). Diabetes prevalence in patients with bradycardiac arrhythmias. *Acta Diabetologica Latina*.

[74] Movahed M.-R., Hashemzadeh M., Jamal M. M. (2005). Increased prevalence of third-degree atrioventricular block in patients with type II diabetes mellitus. *Chest*.

[75] Hillis G. S., Woodward M., Rodgers A. (2012). Resting heart rate and the risk of death and cardiovascular complications in patients with type 2 diabetes mellitus. *Diabetologia*.

[76] Krishnaswamy P. S., Egom E. E., Moghtadaei M. (2015). Altered parasympathetic nervous system regulation of the sinoatrial node in Akita diabetic mice. *Journal of Molecular and Cellular Cardiology*.

[77] Yan B., Li L., Harden S. W. (2009). Diabetes induces neural degeneration in nucleus ambiguus (NA) and attenuates heart rate control in OVE26 mice. *Experimental Neurology*.

[78] Ieda M., Kimura K., Kanazawa H., Fukuda K. (2008). Regulation of cardiac nerves: a new paradigm in the management of sudden cardiac death?. *Current Medicinal Chemistry*.

[79] Shimoni Y. (2001). Inhibition of the formation or action of angiotensin II reverses attenuated K^+^ currents in type 1 and type 2 diabetes. *Journal of Physiology*.

[80] Pahor M., Bernabei R., Sgadari A. (1991). Enalapril prevents cardiac fibrosis and arrhythmias in hypertensive rats. *Hypertension*.

[81] Gay-Jordi G., Guash E., Benito B. (2013). Losartan prevents heart fibrosis induced by long-term intensive exercise in an animal model. *PLoS ONE*.

[82] Bathgate R. A. D., Lekgabe E. D., McGuane J. T. (2008). Adenovirus-mediated delivery of relaxin reverses cardiac fibrosis. *Molecular and Cellular Endocrinology*.

[83] Alves Bento A. S., Bacic D., Saran Carneiro J. (2015). Selective late INa inhibition by GS-458967 exerts parallel suppression of catecholamine-induced hemodynamically significant ventricular tachycardia and T-wave alternans in an intact porcine model. *Heart Rhythm*.

[84] Lehnart S. E., Terrenoire C., Reiken S. (2006). Stabilization of cardiac ryanodine receptor prevents intracellular calcium leak and arrhythmias. *Proceedings of the National Academy of Sciences of the United States of America*.

[85] Xie Y., Liao Z., Grandi E., Shiferaw Y., Bers D. M. (2015). Slow [Na]_i_ changes and positive feedback between membrane potential and [Ca]_i_ underlie intermittent early afterdepolarizations and arrhythmias. *Circulation: Arrhythmia and Electrophysiology*.

[86] Tse G., Tse V., Yeo J. M. (2016). Ventricular anti-arrhythmic effects of heptanol in hypokalaemic, Langendorff-perfused mouse hearts. *Biomedical Reports*.

[87] Tse G., Yeo J. M., Chan Y. W., Lai E. T., Yan B. P. (2016). What is the arrhythmic substrate in viral myocarditis? Insights from clinical and animal studies. *Frontiers in Physiology*.

[88] Tse G. (2016). (Tpeak-Tend)/QRS and (Tpeak-Tend)/(QT x QRS): novel markers for predicting arrhythmic risk in Brugada syndrome. *Europace*.

